# Controllable Synthesis of ZnO Nanoparticles with Improved Photocatalytic Performance for the Degradation of Rhodamine B under Ultraviolet Light Irradiation

**DOI:** 10.3390/molecules28135135

**Published:** 2023-06-30

**Authors:** Xinyue Ren, Yien Du, Xinji Qu, Yumei Li, Luxi Yin, Kaixin Shen, Jingwen Zhang, Yufang Liu

**Affiliations:** 1 Department of Chemistry and Chemical Engineering, Jinzhong University, Jinzhong 030619, China; rxy6268@163.com (X.R.); ylx7627@163.com (L.Y.); skx7883@163.com (K.S.); zjw258@126.com (J.Z.); liuyufang@jzxy.edu.cn (Y.L.); 2 Qingdao Second Health School of Shandong Province, Qingdao 266308, China; 13589200706@163.com

**Keywords:** zinc oxide, semiconductor photocatalyst, photocatalytic activity, cooperative effects

## Abstract

In this work, two-dimensional (2D) Zn-HMT (Zn(NO_3_)_2_(HMT)_2_(H_2_O)_2_]*_n_*) nanosheets were synthesized using a facile one-step chemical precipitation in the presence of Zn(NO_3_)_2_, hexamine (HMT), and anhydrous ethanol at room temperature. Subsequently, hexagonal T*x*-ZnO (T*x*-ZnO refers to the zinc oxide (ZnO) nanoparticles) were synthesized by a high-temperature solid-phase method at different temperatures (*x* = 500, 550, 600, 650, 700, 750, and 800 °C) nanoparticles with different morphologies were synthesized by a high-temperature calcination approach using 2D Zn-HMT nanosheets as precursor. The crystal structure, morphology, specific surface areas, surface and interface properties, optical properties, and charge migration behaviors of the as-synthesized T*x*-ZnO nanoparticles were characterized by powder X-ray diffraction (XRD), field-emission scanning electron microscopy (FESEM), transmission electron microscopy (TEM), high-resolution TEM (HRTEM), automatic specific surface and aperture analyzer, X-ray photoelectron spectroscopy (XPS), UV-visible spectrophotometer, photoluminescence (PL) spectra, and electrochemical impedance spectroscopy (EIS). The photocatalytic performances and stabilities of the as-synthesized typical T*x*-ZnO nanoparticles with various morphologies were evaluated and compared with the commercial ZnO (CM-ZnO) nanoparticle. The T700-ZnO nanoparticle with spherical and irregular morphology exhibited the highest photocatalytic activity (99.12%) for the degradation of Rhodamine B (RhB), compared to T500-ZnO (92.32%), T600-ZnO (90.65%), T800-ZnO (44.04%), and the CM-ZnO (88.38%) nanoparticle, which can be attributed to the cooperative effects of higher crystallinity, bigger crystal size, the strongest separation efficiency, the lowest recombination rate, the fastest charge carrier transfer path, and the highest charge-transfer efficiency. The superior photocatalytic activity illustrated by the T700-ZnO nanoparticle makes it have potential application prospects for the treatment of organic wastewater.

## 1. Introduction

Currently, the design and synthesis of metal oxide semiconductors with various morphologies have potential application prospects in chemical sensing, solar cells, light-emitting diodes, and photocatalytic treatment of wastewater containing persistent organic pollutants [1,2,3,4]. Various semiconductors have been prepared, such as titanium dioxide (TiO_2_), zinc sulfide (ZnS), gallium nitride (GaN), copper oxide (CuO), ferric oxide (Fe_2_O_3_), and zinc oxide (ZnO). Among the semiconductors mentioned above, ZnO is an excellent n-type wide bandgap (3.37 eV) semiconductor that is very suitable to be used as a photocatalyst because of its superior photocatalytic activity, high thermal stability, strong adsorption efficiency, and non-toxicity [4,5,6,7,8]. Generally, ZnO exists in three polymorphs namely: rock salt, wurtzite (hexagonal, space group *P*6_3_*mc*), and zinc blende (cubic, space group *P*a-3). Among the three different crystalline phases of ZnO, hexagonal wurtzite ZnO possesses the highest photocatalytic activity and is widely accepted in catalytic applications. Compared to hexagonal wurtzite ZnO, rock salt ZnO is very rare because it only exists under conditions of high pressure [9]. There are two primary methods for preparing ZnO nanoparticles: gas-phase synthesis and liquid-phase synthesis. Gas-phase synthesis mainly includes gas–liquid–solid growth, chemical vapor deposition, physical vapor deposition, pulsed laser deposition, etc. For example, Song et al. successfully fabricated vertically aligned ZnO nanorods on wide band gap substrates (GaN, Al_0.5_Ga_0.5_N, and AlN) through a vapor–liquid–solid growth method [10]. Chu et al. successfully synthesized well-aligned ZnO nanowires with excellent photocatalytic performance for the degradation of methylene blue on Si(100) substrates through carbothermal reduction and vapor–liquid–solid methods [11]. Protasova et al. synthesized vertically aligned ZnO nanowires on Si substrates by the chemical vapor deposition method. In liquid-phase synthesis, the growth process is carried out in water or organic solutions at relatively low temperatures [12]. Compared to the gas-phase synthesis technique, the liquid-phase synthesis method is more suitable for mass production due to its advantages of low production cost, scalability, and low temperature [12]. For example, Lu et al. successfully prepared ZnO microcrystals with ellipsoidal morphology through a hydrothermal process using zinc nitrate and ammonia as the Zn^2+^ ion and base sources, respectively [13]. Chen et al. successfully synthesized ZnO nanocrystals with various morphologies such as bullet-like, rod-like, sheet-like, polyhedron-like, and crushed stone-like through a hydrothermal process using different organic compounds as template agents [14]. Ta et al. successfully synthesized metal (M)-doped ZnO/g-C_3_N_4_ composites with a sponge-like porous structure by a facile one-pot pyrolysis method using Zn(NO_3_)_2_, Mg(NO_3_)_2,_ and CH_4_N_2_O as the precursors [15]. Ta et al. also successfully synthesized three-level AgNWs/ZnO NRs/AgNPs hierarchical nanostructures via a three-step solution method [16]. Recently, using a deep eutectic solvent as a template, Liu et al. synthesized flower-like ZnO nanoparticles with higher photocatalytic performance for the degradation of methyl orange through a simple hydrothermal method [17]. Benu et al. synthesized fibrous ZnO microrods with higher photocatalytic efficiency for the degradation of RhB through a macroemulsion-mediated solvothermal method using zinc nitrate and zinc acetate as precursors [18]. The detailed synthesis conditions, experimental variables, and morphologies of the various aforementioned strategies are presented in Table 1.

In this work, hexagonal T*x*-ZnO nanoparticles with different morphologies were first synthesized by a facile one-step precipitation method combined with a high-temperature calcination method using zinc nitrate and hexamine as the sources and absolute ethyl alcohol as the solvent. Under heating treatment (500–800 °C), the Zn-HMT precipitate can be decomposed into hexagonal T*x*-ZnO nanoparticles. The crystal phases, morphologies, surface and interface properties, optical properties, and photocatalytic performances of the as-synthesized T*x*-ZnO nanoparticles were explored to elucidate the correlation between their structure and properties. The photocatalytic activity of T*x*-ZnO nanoparticles with various morphologies was studied by the degradation of RhB. Among the four typical photocatalysts calcined at different temperatures, the photocatalytic degradation efficiency increased in the order of T800-ZnO (44.04%) < T600-ZnO (90.65%) < T500-ZnO (92.32%) < T700-ZnO (99.12%). Among them, the T700-ZnO sample is the most effective photocatalyst due to the cooperative effects of higher crystallinity, bigger crystal size, the strongest separation efficiency, the lowest recombination rate, the fastest charge carrier transfer path, and the highest charge-transfer efficiency. The superior photocatalytic activity of T700-ZnO makes it a promising candidate for future photocatalytic applications.

## 2. Results and Discussion

### 2.1. Structural Analysis

**Figure 1:**
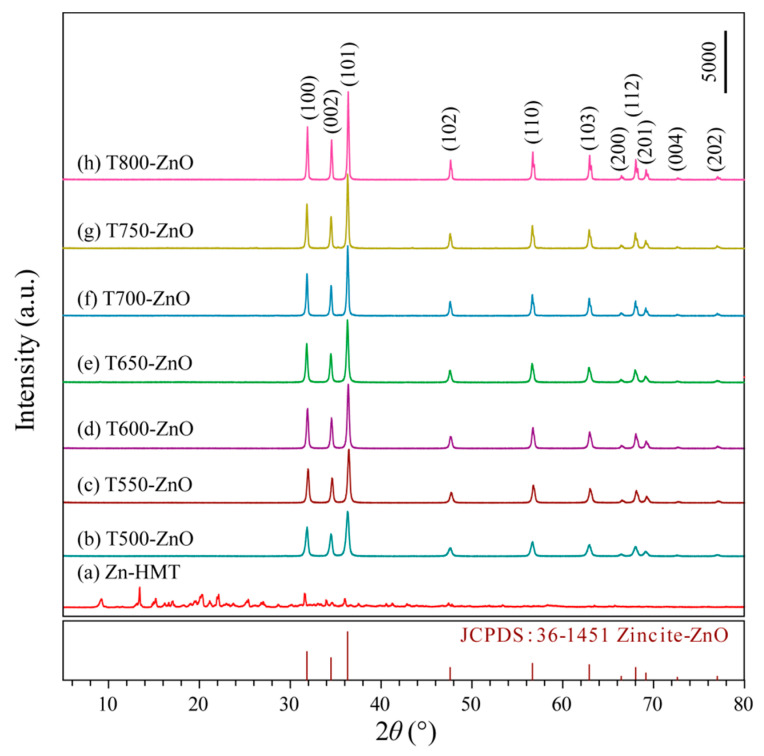
XRD patterns of the (**a**) Zn-HMT precipitation and the as-synthesized (**b**) T500-ZnO, (**c**) T550-ZnO, (**d**) T600-ZnO, (**e**) T650-ZnO, (**f**) T700-ZnO, (**g**) T750-ZnO, and (**h**) T800-ZnO nanoparticles.

After mixing Zn(NO_3_)_2_ and hexamine (HMT) in anhydrous ethanol, a high yield of white precipitation of two-dimensional (2D) Zn-HMT (Zn(NO_3_)_2_(HMT)_2_(H_2_O)_2_]*_n_*) nanosheets can be produced immediately [19], and the corresponding XRD pattern is shown in Figure 1a. The XRD patterns of ZnO samples obtained by calcining the white Zn-HMT precipitation in an air atmosphere of 500~800 °C for 2 h are shown in Figure 1b–h. For all T*x*-ZnO (*x* = 500, 550, 600, 650, 700, 750, 800) samples, only the diffraction peaks of the ZnO phase were observed, indicating that the Zn-HMT precipitation was completely transformed to the ZnO phase at 500~800 °C. The peaks at 2*θ* values of 31.86°, 34.48°, 36.32°, 47.60°, 56.68°, 62.96°, 66.46°, 67.98°, 69.60°, 72.58°, and 77.06° are undisputedly indexed to (100), (002), (101), (102), (110), (103), (200), (112), (201), (004), and (202) crystal planes of hexagonal ZnO (JCPDF no. 36-1451, *P*6_3_*mc*, *a* = *b* = 3.2498 Å, *c* = 5.2066 Å), respectively. The sharp peaks of diffraction observed in the XRD patterns indicate that the as-synthesized T*x*-ZnO nanoparticles are highly crystalline. And the intensity of the diffraction peaks becomes sharper with the increase in calcination temperature, indicating that the crystallinity of the as-prepared T*x*-ZnO nanoparticles increases with the increase in calcination temperature. The average crystallite sizes of the synthesized T500-ZnO, T550-ZnO, T600-ZnO, T650-ZnO, T700-ZnO, T750-ZnO, and T800-ZnO nanoparticles calculated by applying the Scherrer equation (*d* = *kλ*/*β*cos *θ*) [20] to the peak at 2*θ* = 36.32° in the (101) plane are 27.4, 39.1, 45.8, 61.5, 63.8, 81.9, and 90.9 nm, respectively.

### 2.2. Morphological Analysis

**Figure 2:**
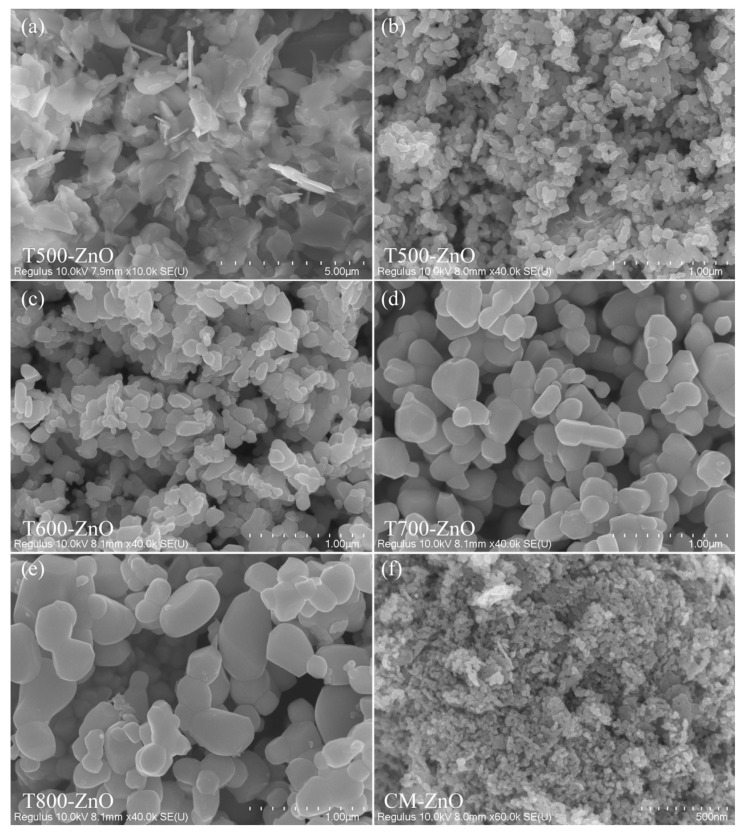
FESEM images of the (**a**) Zn-HMT precipitation and the as-synthesized (**b**) T500-ZnO, (**c**) T600-ZnO, (**d**) T700-ZnO, (**e**) T750-ZnO, and (**f**) CM-ZnO nanoparticles.

The morphologies of the Zn-HMT precipitates and the as-synthesized T*x*-ZnO nanoparticles are studied using the FESEM images (Figure 2). Figure 2a shows a typical FESEM image of the obtained Zn-HMT precipitates with a molar ratio of 1:1. The Zn-HMT precursor presents a two-dimensional, irregular, plate-like structure. After a simple pyrolysis process at 500~800 °C in air, the two-dimensional Zn-HMT plate-like structure is successfully converted to T*x*-ZnO nanoparticles. Figure 2b displays a typical FESEM image of a T500-ZnO sample prepared by calcining the Zn-HMT precursor at 500 °C. As the FESEM images illustrate, most of the T500-ZnO nanoparticles are almost irregularly spherical in morphology, with an average size of 58.6 nm (diameter ranging from 24.5 to 98.3 nm). In addition, some irregular large nanoparticles with a length of 118.5~189.5 nm and some rod-like nanoparticles with a length of 49.6~130 nm and a width of 26.9~56.6 nm were also observed. As can be seen from Figure 2c, when the calcination temperature is 600 °C, many large irregularly shaped nanoparticles with an average size of 164.6 nm (length of about 102~304 nm) and some small rod-shaped, spherical, and irregular morphologies with an average size of 47.5 nm (length of about 25.7~71.9 nm) are observed in the T600-ZnO sample. Figure 2d,e display the typical FESEM images of T700-ZnO and T800-ZnO samples, respectively. As the FESEM images illustrate, most of the T700-ZnO (T800-ZnO) nanoparticles are spherical in morphology with a size of 109.7~238.8 nm (106.1~244.4 nm) in diameter and irregular in morphology with an average size of 259.5 nm (312.2 nm), respectively. Figure 2f displays the FESEM images of the CM-ZnO sample. As can be seen, all of the nanoparticles are found to be approximately 12.7~33.8 nm with a mean size of 21.2 nm. 

### 2.3. Microstructure Analysis

**Figure 3:**
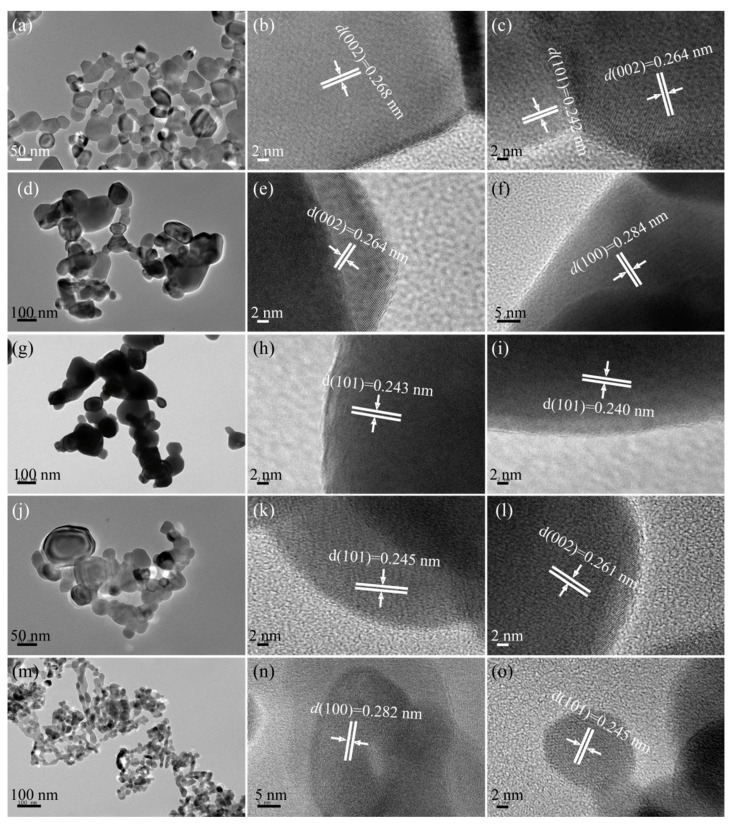
TEM and HRTEM images of the (**a**–**c**) T500-ZnO, (**d**–**f**) T600-ZnO, (**g**–**i**) T700-ZnO, (**j**–**l**) T750-ZnO, and (**m**–**o**) CM-ZnO nanoparticles.

**Figure 4:**
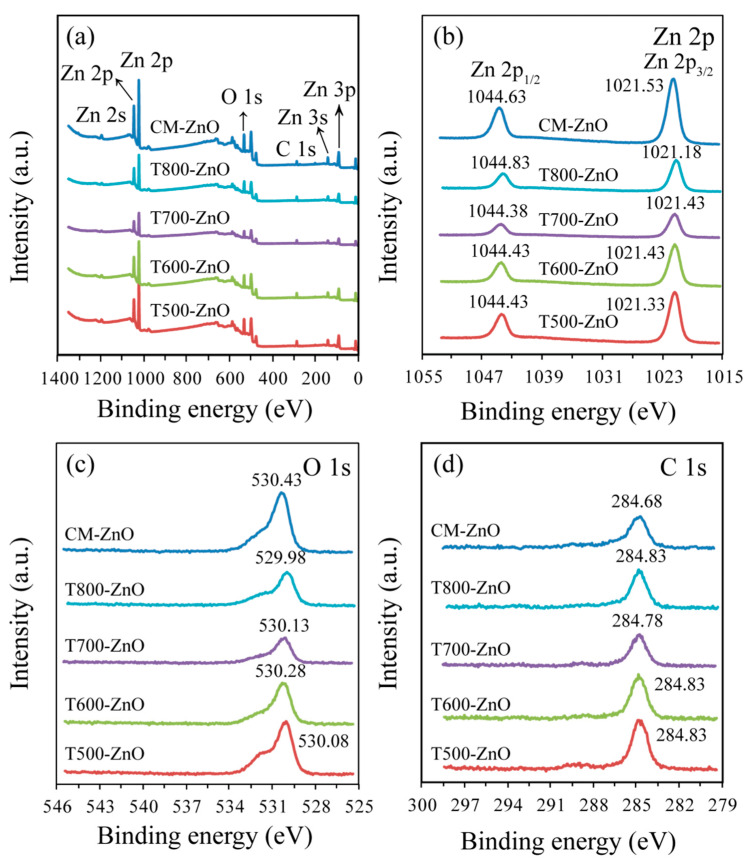
(**a**) Survey and high-resolution XPS spectra of (**b**) Zn 2p, (**c**) O 1s, and (**d**) C 1s for T500-ZnO, T600-ZnO, T700-ZnO, T800-ZnO, and CM-ZnO nanoparticles.

The TEM images of the as-synthesized T*x*-ZnO and CM-ZnO nanoparticles are described below. Figure 3a–c shows the TEM and HRHRTEM images of the as-synthesized T500-ZnO nanoparticles. It can be clearly seen from Figure 3a that the particle size of the synthesized T500-ZnO nanoparticles is in the range of 16.2~96.7 nm, with an average size of 48.1 nm. The ordered lattice spacings of approximately 0.268 (or 0.264) and 0.242 nm correspond to the interplanar distance of the (002) and (101) crystal planes of hexagonal ZnO, respectively (Figure 3b,c). Figure 3d shows the TEM image of a T600-ZnO, which has a particle size of approximately 39.3~207.4 nm (an average size of 97.5 nm). The evident and well-ordered lattice spacings of approximately 0.264 and 0.284 nm correspond to the (002) and (100) crystal planes of hexagonal ZnO (Figure 3e,f). In Figure 3g, the T700-ZnO nanoparticles can be seen with particle sizes ranging from 50.0 nm to less than 196.1 nm (an average size of 102.6 nm). The lattice spacings of 0.243 (or 0.240) nm in the HRTEM images correspond to the (101) crystallographic planes of hexagonal ZnO. Figure 3j–l shows the TEM and HRTEM analysis results of the T800-ZnO sample. The size of spherical and irregular ZnO nanoparticles is about 22.7~42.7 nm in diameter and 37.4~127.0 nm in length, respectively (Figure 3j). And the lattice fringe of 0.245 and 0.261 nm corresponds to the distance between two adjacent (101) and (002) planes of hexagonal ZnO, respectively, as shown in Figure 3k,l. As can be seen from Figure 3m, the CM-ZnO sample is mainly composed of an ellipsoid with a diameter of 13.9~34.4 nm (average size 24.5 nm), nanorods with approximately 19.0~68.8 nm in length (average size 35.4 nm), and a rhombus with 29.0~45.9 nm in a diagonal line. From Figure 4n,o, the lattice fringes with interplanar distances of 0.282 and 0.245 nm can be observed, corresponding to the (100) and (101) crystal planes of the hexagonal ZnO, respectively.

### 2.4. Surface and Interface Analysis

To further reveal the composition of the samples, the surface properties of the as-prepared typical T*x*-ZnO and CM-ZnO samples were investigated using X-ray photoelectron spectroscopy (XPS). Gaussian deconvolution of the high-resolution spectra was used to distinguish different types of chemical bonds, especially the chemical states of elements in the as-prepared T*x*-ZnO and CM-ZnO samples. As shown in the survey spectra of Figure 4a, the T*x*-ZnO and CM-ZnO samples only display emissions of Zn and O elements with only a weak C line, with the different peaks observed being assigned to Zn 2s, Zn 2p, Zn 3s, Zn 3p, and O 1s core levels and to Zn *LMM* and O *KLL* Auger features [21]. The high-resolution XPS for Zn 2p (Figure 4b) reveals the spin orbital splitting of the Zn 2p_1/2_ and Zn 2p_3/2_ core level states of zinc centered at 1044.43~1044.83 eV and 1021.18~1021.53 eV, respectively, which were symmetric and were assigned to lattice zinc oxide. The separation between the Zn 2p_1/2_ and Zn 2p_3/2_ levels (22.95 (1044.38–1021.43)~23.1 eV (1044.63–1021.53)) corresponds to the spectrum of ZnO reported in the literature [21,22]. The measured emission lines also correspond to the normal oxidation state of the Zn^2+^ ion in ZnO. The corresponding peaks in T500-ZnO, T600-ZnO, and T700-ZnO shifted to lower binding energies, indicating that there is a strong interaction between ZnO [23]. As for the O 1s spectrum (Figure 4c), the major peaks at 529.98~530.43 eV can be attributed to lattice oxygen in the ZnO (i.e., Zn–O). For the C 1s spectrum (Figure 4d), the major peaks at 284.68~284.83 eV are assigned to the residual carbon and adventitious hydrocarbons absorbed on the surface of ZnO, corresponding to the C–C or C–H bonds [22].

### 2.5. Optical Properties

**Figure 5:**
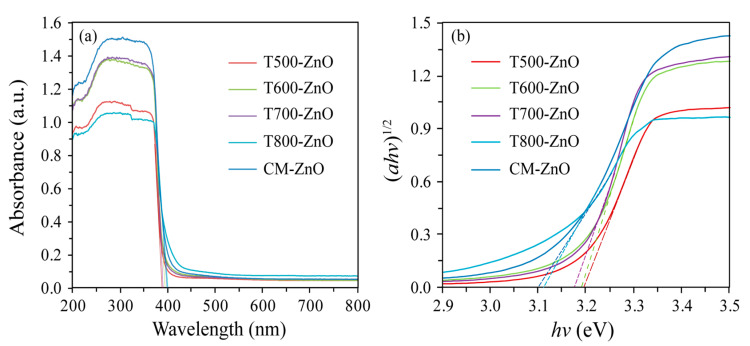
(**a**) UV-Vis diffuse reflection spectra and (**b**) Kubelka–Munk function (*ahv*)^1/2^ as a function of photon energy (*hv*) of the T500-ZnO, T600-ZnO, T700-ZnO, T800-ZnO, and CM-ZnO nanoparticles.

The optical properties of the synthesized T*x*-ZnO and CM-ZnO samples were investigated by UV-vis diffuse reflection spectra and photoluminescence (PL). As shown in Figure 5a, all samples illustrated a prominent absorption edge and an Urbach resembling absorption tail in the range of 200–800 nm. Using the concept of the edge at the intersection of wavelengths through extrapolation of the horizontal and sharply rising portions of the curves, the absorption edges of the T500-ZnO, T600-ZnO, T700-ZnO, T800-ZnO, and CM-ZnO nanoparticles were estimated to be about 388, 387, 385, 400, and 396 nm, respectively. The optical band gap energies (*E*_g_) of the T500-ZnO, T600-ZnO, T700-ZnO, T800-ZnO, and CM-ZnO nanoparticles can be calculated as 3.19, 3.20, 3.22, 3.10, and 3.13 eV, respectively, according to the formula *Band gap* = 1240/*Wave length* [24]. Using the Kubelka–Munk function (*ahv*)^1/2^ (where *a* is the absorption coefficient) as a function of photon energy (*hv*) [25], it is calculated that the *E*_g_ of the T500-ZnO, T600-ZnO, T700-ZnO, T800-ZnO, and CM-ZnO nanoparticles was 3.19, 3.17, 3.19, 3.11, and 3.14, respectively, as shown in Figure 5b. The values of band gap energies were in good agreement with those reported in other literature [21]. The absorption intensity of the as-synthesized samples changed with the calcination temperatures, increasing in the order of T800-ZnO < T500-ZnO < T600-ZnO < T700-ZnO < CM-ZnO.

**Figure 6:**
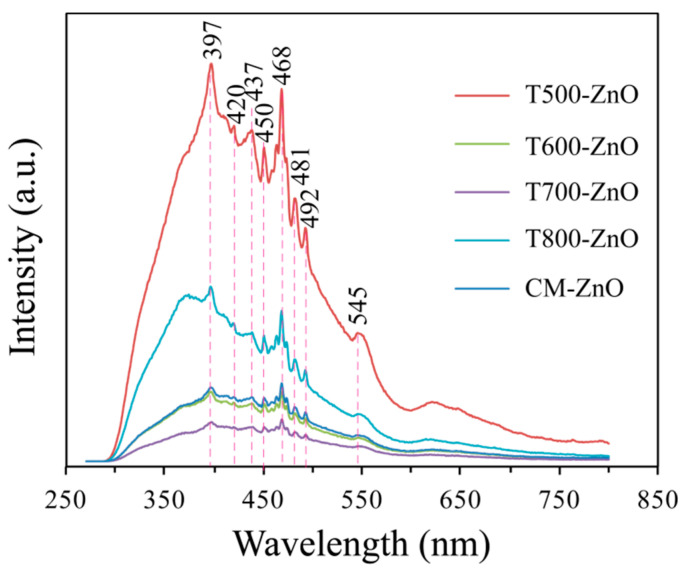
PL emission spectra of the T500-ZnO, T600-ZnO, T700-ZnO, T800-ZnO, and CM-ZnO nanoparticles.

Photoluminescence (PL) analysis can effectively reveal the optical and photochemical properties of ZnO. Information related to crystal quality, structural defects (surface oxygen vacancies, zinc interstitials, etc.), and particle surfaces can be obtained from PL spectra [26]. The room-temperature PL emission spectra (excitation wavelength was 325 nm) of the synthesized T*x*-ZnO samples calcined at 500~800 °C and the CM-ZnO sample are shown in Figure 6. The PL spectra of all the synthesized T*x*-ZnO samples and the CM-ZnO sample illustrate five sharp peaks at about 397, 450, 468, 481, and 492 nm and three broad peaks at about 420, 437, and 545 nm. However, various peak intensities are achieved for T*x*-ZnO particles prepared at different temperatures and CM-ZnO. The sharp peak located at about 397 nm in the UV region corresponds to the excitonic emission, which is a near-band-edge emission [26]. The peaks located at about 420 nm and 481 nm in the blue band region correspond to band edge free excitons and bound excitons, respectively [26]. The peak located at about 437 nm in the blue band region can be attributed to the presence of an oxygen vacancy in the ZnO lattice, which is caused by the recombination of radiating electron holes in the oxygen vacancy sites [20]. The peak located at near 545 nm in the green region is associated with surface recombination and singly ionized oxygen vacancies [27]. In PL spectroscopy, the intensity of the peak is related to the recombination and separation of electrons and holes. Generally speaking, a high-intensity peak indicates fast recombination of electrons and holes, while a low-intensity peak indicates better separation of electrons and holes [20]. From Figure 6, it is evident that the peak intensity decreases in the order T500-ZnO > T800-ZnO > CM-ZnO > T600-ZnO > T700-ZnO; that is, the T700-ZnO sample displays the most efficient charge carrier separation and enhanced photocatalytic performance.

### 2.6. Electrochemical Impedance Spectroscopy Analysis

**Figure 7:**
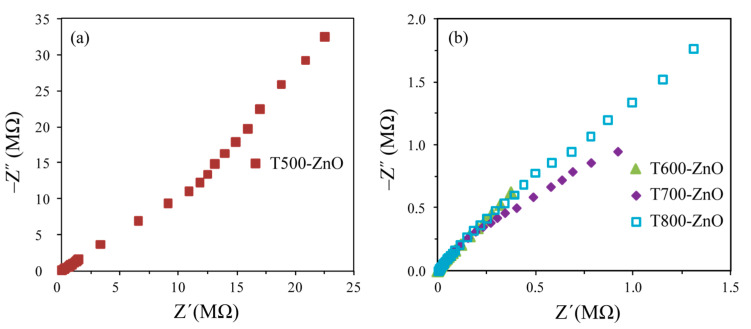
EIS Nyquist plots of the (**a**) T500-ZnO, (**b**) T600-ZnO, T700-ZnO, and T800-ZnO nanoparticles.

Electrochemical impedance spectroscopy (EIS) was carried out to understand the separation capability of photoinduced electron–hole pairs. Figure 7 shows the Nyquist plots of the T500-ZnO, T600-ZnO, T700-ZnO, and T800-ZnO nanoparticles obtained at an AC amplitude of 0.01 V in the frequency range of 0.1 Hz to 0.1 MHz. It has been reported that a smaller arc radius in the Nyquist plot means more efficient separation of photoinduced electron–hole pairs and faster transfer of the interfacial charge between electrolyte and semiconductor [28]. It can be seen that the order of the impedance arc radius of the nanoparticles is T700-ZnO < T600-ZnO < T800-ZnO < T500-ZnO, indicating that the T700-ZnO nanoparticle has a faster charge carrier transfer path to the electron acceptor and a higher charge-transfer efficiency, which is conducive to the improvement of photocatalytic activity [29].

### 2.7. Photocatalytic Properties

**Figure 8:**
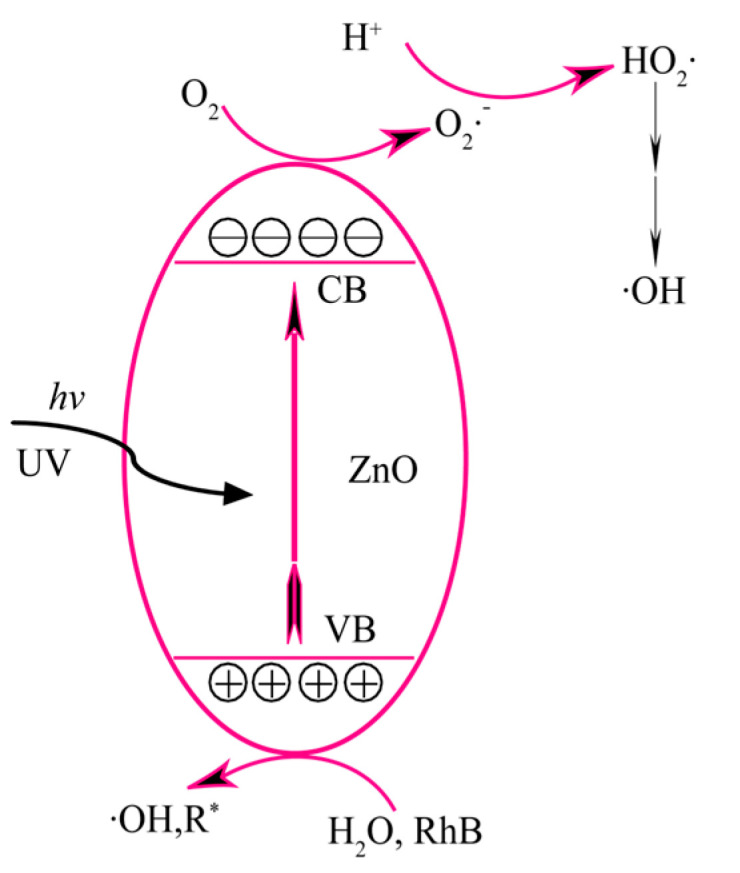
Schematic of mechanism of photocatalysis on ZnO.

The relative photocatalytic activity of the T*x*-ZnO nanoparticles synthesized at different temperatures was evaluated by monitoring the concentration changes in RhB solution during the photocatalysis. Figure 8 illustrates a possible mechanism for the main photocatalytic reactions on the surface of ZnO nanoparticles [30,31]. Usually, when the energy of photons (*hν*) from UV light irradiation is equal to or greater than the band gap energy of the ZnO nanoparticles, numerous electrons (e^−^) on the valence band (VB) of the ZnO nanoparticles will be excited into the conduction band (CB), leaving an equal amount of holes (h^+^) on the conduction band (Equation (1)). The photogenerated holes in the VB with strong oxidizability can oxidize water (H_2_O) molecules (or hydroxyl groups (OH^−^)) and RhB molecules adsorbed on the surface of ZnO nanoparticles to hydroxyl radicals (·OH) and R^*^ radicals (Equations (2)–(4)). Meanwhile, the photogenerated electrons with strong reducibility can reduce oxygen (O_2_) molecules adsorbed on the surface of ZnO nanoparticles to superoxide ion radicals (O_2_·^−^) (Equation (5)) and hydrogen peroxide radicals (HO_2_·) (Equation (6)). More ·OH radicals can also be formed by the reaction of the HO_2_· radical with the trapped electron (Equations (7) and (8)). The active oxygen species O_2_·^−^, HO_2_·, or ·OH radicals, especially the ·OH radical, can oxidize the RhB dye (or the corresponding R* radical) to mineralized products (Equation (9)). The above photocatalytic process can be described as follows [30,31]:

ZnO nanoparticles + *hν* → h^+^ + e^−^ (1)

h^+^ + H_2_O → ·OH + H^+^ (2)

h^+^ + OH^−^→ ·OH (3)

h^+^ + RhB → R* (4)

e^−^ + O_2_ → O_2_·^−^ (5)

H^+^ + O_2_·^−^ → HO_2_ (6)

e^−^ + HO_2_· + H^+^ → H_2_O_2_ (7)

H_2_O_2_ + e^−^ →·OH + OH^−^ (8)

RhB or R* + { O_2_·^−^, HO_2_·, or ·OH} →CO_2_ + H_2_O + other degradation products (9)

**Figure 9:**
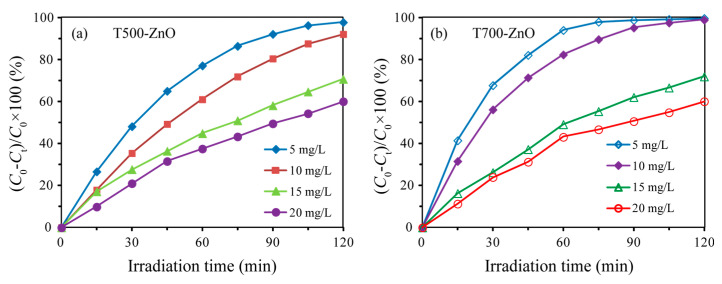
Photoactivities of (**a**) T500-ZnO and (**b**) T700-ZnO samples for RhB solutions with different concentrations.

The photocatalytic degradation efficiency (*η*) of RhB with different concentrations in the presence of T500-ZnO and T700-ZnO nanoparticles was calculated using Equation: *η* = (*C*_0_ − *C*_t_)/*C*_0_ × 100% [32], and is shown in Figure 9a,b, respectively. For T500-ZnO nanoparticles, after 120 min of UV light irradiation, the percentage of RhB with different concentrations of degradation was as follows: 5 mg/L (97.96%), 10 mg/L (92.32%), 15 mg/L (70.84%), and 20 mg/L (60.19%). For T700-ZnO nanoparticles, after 120 min of UV light irradiation, the percentage of RhB with different concentrations of degradation was as follows: 5 mg/L (99.88%), 10 mg/L (99.12%), 15 mg/L (72.08%), and 20 mg/L (60.21%). The above results show that the degradation efficiency of RhB solution is inversely proportional to its concentration under the same conditions.

**Figure 10:**
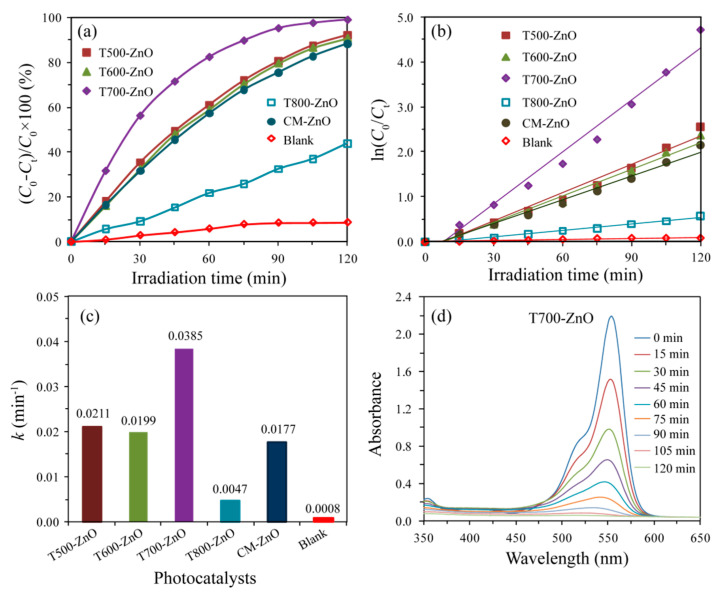
(**a**) Photoactivities of the T500-ZnO, T600-ZnO, T700-ZnO, T800-ZnO, CM-ZnO, and Blank samples for RhB degradation, (**b**,**c**) corresponding pseudo-first-order dynamics and apparent rate constants, (**d**) temporal evolution of RhB absorption over T700-ZnO nanoparticle.

**Figure 11:**
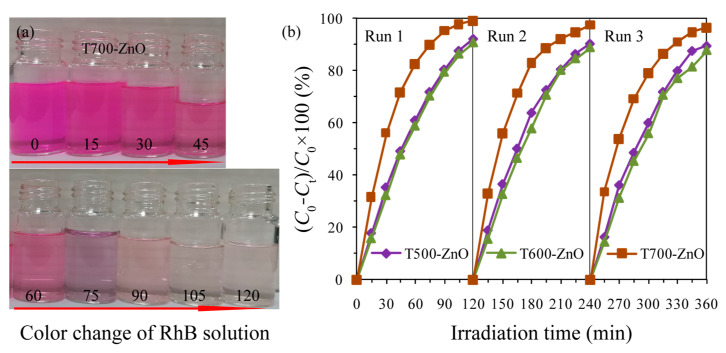
(**a**) Color variation of RhB solution with illumination time in the presence of T700-ZnO sample; (**b**) Cycling runs of the 150 mL 10 mg/L RhB aqueous in the presence of 0.10 g T500-ZnO, T600-ZnO, and T700-ZnO samples, respectively.

Figure 10a shows the photocatalytic degradation curves of RhB solution (10 mg/L) over the as-synthesized T*x*-ZnO (*x* = 500, 600, 700, 800), CM-ZnO, and the blank samples. The self-photodegradation efficiency of the RhB is only 8.66% without a ZnO sample, while T700-ZnO shows the most efficient degradation efficiency (99.12%), followed by T500-ZnO (92.32%), T600-ZnO (90.65%), CM-ZnO (88.38%), and T800-ZnO (44.04%). The corresponding pseudo-first-order kinetic plots and apparent rate constants (*k*, min^−1^) were obtained by substituting the photocatalytic experiment data into −ln(*C*_t_/*C*_0_) = *kt* [32], as shown in Figure 10b,c, respectively. The *k* values are 0.0211, 0.0199, 0.0385, 0.0047, 0.0177, and 0.0008 min^−1^ for T500-ZnO, T600-ZnO, T700-ZnO, T800-ZnO, CM-ZnO, and the blank sample, respectively (Figure 10c). In particular, T700-ZnO gave the highest apparent rate constant of 0.0385 min^−1^, magnified by 1.82, 1.93, 8.19, 2.18, and 48.13 folds as compared to T500-ZnO, T600-ZnO, T700-ZnO, T800-ZnO, CM-ZnO, and the blank sample, respectively. To explore the intrinsic photoactivity, the apparent reaction rate constants (*k*) were also normalized to the specific surface areas (*k*_s_) [33]. The specific surface areas were calculated to be 39.98, 23.92, 17.17, 12.21, and 54.78 m^2^/g for T500-ZnO, T600-ZnO, T700-ZnO, T800-ZnO and CM-ZnO, respectively. The T700-ZnO exhibits the greatest photoactivity with *k*_s_ = 2.21 × 10^−3^ min^−1^·g/m^2^, while *k*_s_ is 5.28 × 10^−4^, 8.39 × 10^−4^, 3.84 × 10^−4^, and 3.23 × 10^−4^ min^−1^·g/m^2^ for T500-ZnO, T600-ZnO, T800-ZnO, and CM-ZnO, respectively. The above analysis results indicate that T700-ZnO exhibits the most superior photocatalytic activity compared to other T*x*-ZnO (*x* = 500, 600, 800) and CM-ZnO samples. The vivid temporal evolution of the RhB adsorption spectrum over T700-ZnO is shown in Figure 10d. With the extension of UV irradiation time, the position of the maximum absorption peak of RhB solution gradually moved to the short wavelength direction (from 554 nm to 521 nm), and the intensity gradually weakened until it disappeared, indicating the complete N-deethylation of RhB and the destruction of polycyclic aromatic hydrocarbon structure [34]. Figure 11a shows the color variation of RhB solution with illumination time in the presence of a T700-ZnO sample under UV irradiation. It can be seen that with the extension of illumination time, the pink color of the RhB solution gradually becomes lighter until it becomes colorless after 120 min. The above results indicate that RhB dye underwent permanent mineralization during photolysis in the presence of T700-ZnO [35].

It is well known that crystal morphology, crystallinity, grain size, specific surface area, generation and separation of charge carriers, and other factors can have an important effect on photocatalytic performance [36]. According to the previous analysis, the particle sizes increase in the order of CM-ZnO (~20 nm) < T500-ZnO (27.4 nm) < T600-ZnO (45.8 nm) < T700-ZnO (63.8 nm) < T800-ZnO (90.9 nm). Generally speaking, the specific surface area is inversely proportional to the size of the crystallite. The specific surface areas increase in the order of T800-ZnO (12.21 m^2^/g) < T700-ZnO (17.17 m^2^/g) < T600-ZnO (23.92 m^2^/g) < T500-ZnO (39.98 m^2^/g) < CM-ZnO (54.78 m^2^/g). Generally speaking, a high specific surface area helps to enrich more organic pollutant molecules on the surface of the photocatalyst, thereby accelerating the progress of photocatalytic reactions [37]. However, the photocatalytic performance of T700-ZnO (99.12%) is 1.07, 1.09, 2.25, and 1.12 times greater than that of T500-ZnO (92.32%), T600-ZnO (90.65%), T800-ZnO (44.04%), and CM-ZnO (88.38%), respectively, indicating that the specific surface area is not an important factor affecting the photocatalytic performance. According to the previous XRD analysis, it has been known that the crystallinity of the synthesized ZnO nanoparticles is directly proportional to the calcination temperature. That is, the crystallinity of the as-synthesized T*x*-ZnO increases in the order of T500-ZnO < T600-ZnO < T700-ZnO < T800-ZnO. As we all know, the improvement of crystallinity can reduce the surface defects in the sample, which is beneficial for increasing the migration of photogenerated carriers, reducing the recombination sites of electron–hole pairs, and improving the progress of photocatalytic reactions [38]. Therefore, good crystallinity is an important requirement for the high photocatalytic activity of T700-ZnO nanoparticles. Moreover, according to the previous PL and EIS analysis, the peak intensity and the impedance arc radius both decrease in the order T500-ZnO > T800-ZnO > CM-ZnO > T600-ZnO > T700-ZnO, indicating that the T700-ZnO nanoparticle owns the strongest separation efficiency, the lowest recombination rate, the fastest charge carrier transfer path, and the highest charge-transfer efficiency, which is favorable for the enhancement of photocatalytic performance. From the discussion above, it can be concluded that T700-ZnO nanoparticles exhibit the highest photocatalytic activity, which can be attributed to the cooperative effects of higher crystallinity, bigger crystal size, the strongest separation efficiency, the lowest recombination rate, the fastest charge carrier transfer path, and the highest charge-transfer efficiency. It is reported in the literature that ZnO nanoparticles prepared under different conditions had high photocatalytic performance for the degradation of RhB. Table 2 shows the comparison of the catalytic performance of ZnO nanoparticles prepared under different conditions and the corresponding references. It can be seen from the comparison results in Table 2 that T500-ZnO, T600-ZnO, and T700-ZnO nanoparticles prepared in this study have higher catalytic performance for the degradation of RhB dye at room temperature.

**Figure 12:**
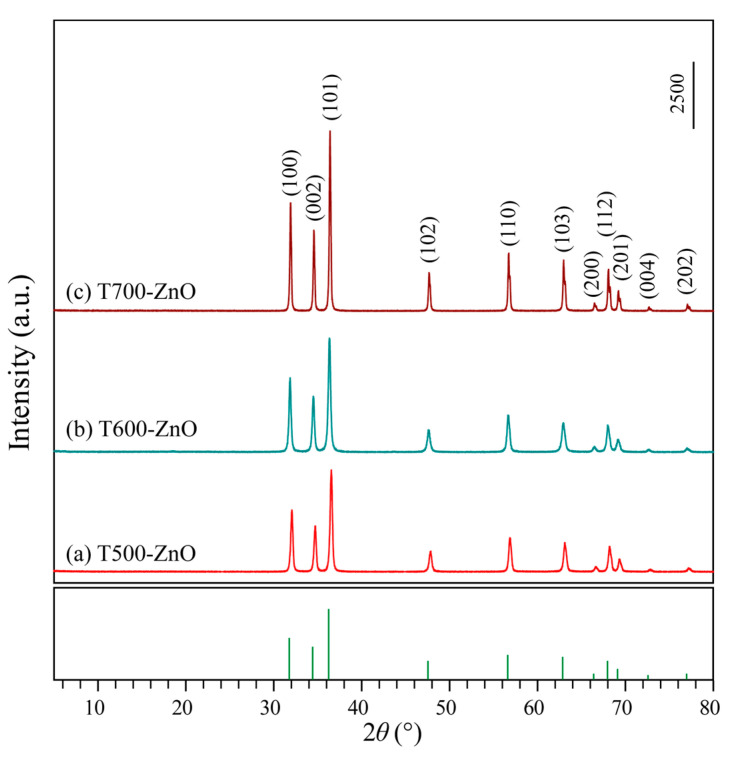
XRD patterns of (**a**) T500-ZnO, (**b**) T600-ZnO, and (**c**) T700-ZnO nanoparticles after three photocatalytic experiments.

The stability of the photocatalyst is a key requirement for its possible practical application. It is well known that ZnO photocatalysts are easily photo-corroded under UV light irradiation, which affects their photocatalytic activity [22]. Hence, it is very important to study the photostability of prepared ZnO. To investigate the stability of the T*x*-ZnO, T500-ZnO, T600-ZnO, and T700-ZnO samples were collected and reused in the three consecutive RhB degradation experiments. The experiment was carried out by adding used T500-ZnO, T600-ZnO, and T700-ZnO samples to fresh RhB solutions with the same concentration (10 mg/L RhB in distilled water). As shown in Figure 11b, after three consecutive cycles to degrade RhB, the degradation efficiency of RhB over pristine T500-ZnO, T600-ZnO, and T700-ZnO samples decayed by only 2.69%, 2.92%, and 2.67%, respectively. Moreover, the XRD patterns of the T500-ZnO, T600-ZnO, and T700-ZnO samples after three experiments are basically the same as those of the fresh samples (Figure 12), indicating that the T500-ZnO, T600-ZnO, and T700-ZnO samples are quite stable in chemical properties. 

## 3. Materials and Methods

### 3.1. Materials

Zinc nitrate (Zn(NO_3_)_2_·6H_2_O, 99.6%, Tianjin Bodi Chemical Co. Ltd., Tianjin, China), hexamine (HMT, 99.0%, Tianjin Guangfu Technology Development Co., Ltd., Tianjin, China), absolute ethyl alcohol (99.7%, Tianjin Kemi Ou Chemical Reagent Co., Ltd., Tianjin, China), and commercial ZnO (CM-ZnO, average size ~20 nm, 99.0%, Sinopharm Chemical Reagent Co., Ltd., Shanghai, China). All chemicals were used as received from the supplier without further purification.

### 3.2. Synthesis of ZnO Nanoparticles

**Figure 13:**
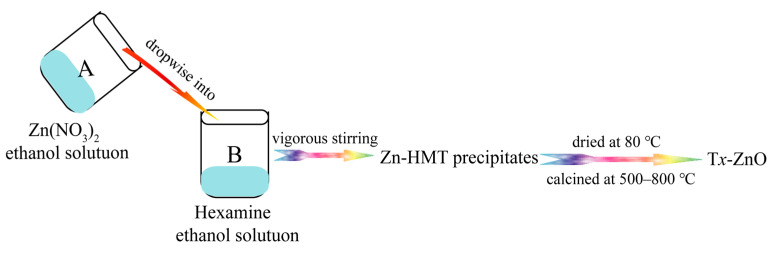
Schematic diagram of synthesis of zinc oxide nanoparticles.

ZnO nanoparticles were synthesized through a facile precipitation method combined with a high-temperature calcination process. In a typical procedure, 21.2728 g of Zn(NO_3_)_2_·6H_2_O was added to 300 mL of anhydrous ethanol under magnetic stirring to obtain a transparent solution A. Meanwhile, 10.1904 g of HMT was dissolved in 500 mL of anhydrous ethanol under magnetic stirring to form a homogeneous solution B. After that, the solution A was subsequently added dropwise into the solution B slowly under vigorous stirring to produce white precipitates immediately, expressed as Zn-HMT. Afterward, the Zn-HMT precipitates were collected by vacuum filtration, washed with absolute ethanol several times, and finally oven dried in air at 80 °C overnight. Finally, the white Zn-HMT precipitates were directly calcined at 500–800 °C for 2 h in an air atmosphere at a heating rate of 2 °C min^−1^, and the corresponding final products were named T*x*-ZnO (*x* represents the calcination temperature of Zn-HMT precipitates, *x* = 500, 550, 600, 650, 700, 750, 800). The synthesis diagram of zinc oxide nanoparticles is shown in Figure 13.

### 3.3. Characterization

The phase composition of the T*x*-ZnO samples was determined by XRD on a Shimadzu XRD-6100 X-ray diffractometer with monochromatic Cu Kα radiation (λ = 0.15406 nm). The accelerating voltage and applied current were 40 kV and 30 mA, respectively. Field-emission scanning electron microscopy (FESEM) images were obtained by the Hitachi SU8100 field-emission transmission electron microscope (Tokyo, Japan) with an accelerating voltage of 30 kV. Transmission electron microscopy (TEM) and high-resolution TEM (HRTEM) images were obtained by a FEI TALOS 200S instrument (Portland, Oregon, America) with an accelerating voltage of 200 kV. The sample was prepared by dropping the sample suspension dropwise onto Formvar/carbon-coated 400 mesh copper grids (FCF400-CU, Electron Microscopy Sciences, Munich, Germany). The Brunauer–Emmett–Teller surface areas were obtained by using a micromeritics ASAP 2020 automatic specific surface and aperture analyzer (Micromeritics Instrument Corp., Atlanta, GA, USA) at 77 K. X-ray photoelectron spectroscopy (XPS) was recorded on a Thermo Fisher Scientific ESCALAB 250XI XPS spectrometer (New York, NY, USA) using monochromatized Al K*α* radiation (1486.6 eV) with a 30 eV pass energy at a 0.05 eV step over a 700 µm × 300 µm sample area. The diffuse reflectance absorption spectra of the samples were measured between 200 and 800 nm with a Shimadzu UV-visible spectrophotometer (UV-2600, Kyoto, Japan) equipped with an integrated sphere attachment. Photoluminescence (PL) spectra were recorded on a HORIBA Fluoromax-4 fluorescence spectrometer (HORIBA Instruments Inc., Kyoto, Japan) with an excitation of 325 nm laser light. Electrochemical impedance spectroscopy (EIS) measurements were performed on a CHI600E electrochemical workstation (Shanghai Chenhua Instrument Co., Ltd., Shanghai, China). An indium–tin oxide glass was used as a working electrode; a platinum plate (opening area: 1 cm^2^) and an Ag/AgCl (saturated KCl solution) were used as a counter electrode and a reference electrode, respectively.

### 3.4. Photocatalytic Activity

**Figure 14:**
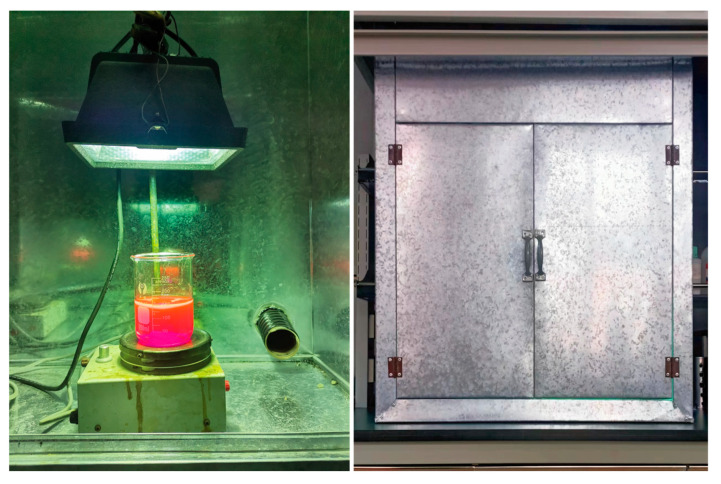
Self-made photocatalytic experimental apparatus.

The photocatalytic reaction was conducted in a self-made photocatalytic experimental apparatus, as shown in Figure 14. A 175 W low-pressure mercury lamp (*λ*_max_ = 365 nm, Shanghai Mingyao Glass Hardware Tool Factory, Shanghai, China) placed in the self-made white iron case was used as the ultraviolet light source, and the door of the white iron case was closed during the photocatalytic experiment. Photocatalytic activities of the as-synthesized T*x*-ZnO and CM-ZnO nanoparticles were evaluated by using Rhodamine B (RhB) as a model organic dye pollutant compound under UV light irradiation. In a typical photodegradation experiment, either T*x*-ZnO or CM-ZnO nanoparticles (0.100 g) were added to an aqueous solution of RhB (150 mL, 5~20 mg/L) to form a suspension in a 250 mL quartz beaker. Prior to the irradiation, the suspensions containing ZnO nanoparticles were magnetically stirred in the dark for 30 min to make RhB molecules reach the absorption–desorption equilibrium on the surface of ZnO nanoparticles. After that, the suspensions were continuously illuminated for 120 min. Under ambient conditions, the initial liquid level of all suspensions in the quartz beaker is 25 cm away from the UV source. At each irradiation interval of 15 min, 4 mL of the aliquots were extracted, centrifuged, and analyzed by recording variations in the absorption band (553 nm) in the UV-visible spectra of RhB using a TU-1901 UV-vis spectrophotometer (Beijing Purkinje General Instrument Co., Ltd., Beijing, China). The degradation ratio of RhB at each time interval was calculated from the ratio of the concentration of the solution irradiated to that nonirradiated.

## 4. Conclusions

ZnO nanoparticles with various morphologies were successfully synthesized via a high-temperature calcination method using 2D Zn-HMT nanosheets as the precursor. XRD, FESEM, TEM, HRTEM, UV-Vis diffuse reflection spectra, PL emission spectra, and EIS measurements were employed to investigate the structure, morphology, microstructure, optical properties, and charge migration behaviors. With the change in calcinations, the band gap of the T*x*-ZnO was enlarged from 3.10 to 3.22 eV. The T700-ZnO nanoparticle with spherical and irregular morphology exhibited the most superior photocatalytic activity for the degradation of RhB, compared to T500-ZnO, T600-ZnO, T800-ZnO, and the benchmark CM-ZnO nanoparticle. The enhanced photocatalytic activity of T700-ZnO nanoparticles can be attributed to the cooperative effects of higher crystallinity, bigger crystal size, the strongest separation efficiency, the lowest recombination rate, the fastest charge carrier transfer path, and the highest charge-transfer efficiency. Our results open a new window for the synthesis of ZnO nanoparticles, which have a high potential for removing organic contaminants from wastewater under irradiation.

## Figures and Tables

**Table 1:** Chemical methods of synthesis of ZnO.

Chemical Methods of Synthesis	Precursors	Synthesis Conditions	Morphologies	Reference
High-temperature solid-phase synthesis method	Zinc acetate dehydrate, sodium lauryl sulfate, sodium hydroxide	Calcined at 580 °C for 2 h	Nanoparticles	[1]
Reflux method	Anhydrous zinc acetate, benzylamine, dibenzyl ether	Refluxed at 220 °C for 5 h; refluxed at 210 °C, 2 h	Nanorods, nanobullets, nanolates	[2]
Hydrothermal method	Zinc nitrate hexahydrate, hexamethylenetetramine	Hydrothermal process at 140 °C for 4 h	Nanorods	[6]
Thermal oxidation procedure	Zn foils, isopropyl alcohol	Zinc foils were thermally oxidized at 520 °C for 60 min, subsequently, samples were heated at 620 °C for 60 min	Nanowires	[8]
Carbothermal reduction and vapor–liquid– solid method.	ZnO, graphite powder,	Calcined ZnO and graphite powder at 950 °C	Nanowires	[11]
Chemical vapor deposition method	Diethylzinc, Zn powder	Heated Zn powder and ZnO seed layer at 650 °C under a constant flow of argon gas	Nanowires	[12]
Hydrothermal method	Zinc nitrate, ammonia	Hydrothermal process at 100–200 °C for 0.5–2 h	Ellipsoidal shape and rod-like shape	[13]
Hydrothermal method	Zinc chloride, sodium hydroxide	Hydrothermal process at 100–220 °C for 5–10 h	Bullet-like, rod-like, sheet, polyhedron, crushed stone-like	[14]
Hydrothermal method	Zinc acetate, sodium hydroxide	Hydrothermal process at 140–170 °C for 4–15 h	Flower-like, cauliflower-like	[17]
Solvothermal method	Zinc nitrate hexahydrate, zinc acetate dehydrate, urea	Solvothermal process at 120 °C for 24 h, then calcined at 400 °C for 4 h	Fibrous microrods	[18]

**Table 2:** Comparison of catalytic performance of ZnO nanoparticles.

Chemical Methods of Synthesis	Raw Materials	Test Conditions	Degradation Efficiency	Reference
Solvothermal treatment, 120 °C for 24 h, then calcined at 400 °C for 4 h	Zinc nitrate hexahydrate, zinc acetate dehydrate, urea	0.0080 g fibrous ZnO microrods, 8 mL 5 ppm RhB aqueous, UV LED	73.82% (60 min)	[18]
Hydrothermal method, 175 °C, 12 h	Stearic acid, Zinc stearate, distilled water	0.100 g ZnO microdishes, 250 mL 1.04 × 10^−5^ mol/L RhB aqueous solution, 300 W mercury lamp	57.1% (90 min)	[39]
Hydrothermal method, 150 °C, 12 h	Zn(NO_3_)_2_·6(H_2_O), Na_2_CO_3_, hexamethylenetetramine,	0.100 g ZnO nanosheets, 40 mg/L RhB aqueous solution, unspecified volume of Rhodamine B solution, 500 W mercury lamp,	96.8% (120 min)	[40]
Hydrothermal method, 200 °C, 8 h	Sesame oil, and urea, zinc acetate dehydrate, ammonia	0.050 g ZnO nanoparticles, 100 mL 10 mg/L RhB aqueous solution, 100 μL 30% H_2_O_2_, unspecified intensity of ultraviolet light source	44% (180 min)	[41]
Sonication, 80 °C, 2 h	zinc acetate dehydrate, sodium hydroxide, distilled water	0.0060 g ZnO nanoparticles, 10 mL 1.0 × 10^−5^ mol/L RhB aqueous solution, 400 W halogen lamp	43% (300 min)	[42]
Low-temperature solution method, 30 °C, 6 days	Zn(NO_3_)_2_·6(H_2_O), distilled water, AgNWs, NaOH, ethyl alcohol, EDA	0.0200 g ZnO nanorods, 50 mL 1.0 × 10^−5^ mol/L RhB aqueous solution, an air mass 1.5 (AM 1.5) solar simulator, the distance from the solar simulator to the reactor was 20 cm,	68.4% (40 min)	[16]
Green Synthesis	Zn(NO_3_)_2_·6(H_2_O) capsicum annuum var	0.0500 g ZnO nanoparticles, 50 mL 15 mg/L RhB aqueous solution, 10 W UV light lamps	92% (180 min)	[43]
Green Synthesis	Zinc acetate dihydrate, methanol, *U. dioica* leaf extract	0.100 g ZnO, 100 mL 10 mg/L RhB aqueous solution, 250 W UV-A lamps	85% (140 min)	[44]
Refluxed, 70 °C, 2.5 h	Zinc acetate dehydrate, methanol, Lithium hydroxide	0.050 g flower-like ZnO, 100 mL 10 ppm RhB aqueous solution, 30 W UV lamp	73% (5 h)	[45]
Precipitation method combined with high-temperature calcination process	Zn(NO_3_)_2_·6H_2_O,HMT, anhydrous ethanol	0.100 g T500-ZnO nanoparticles, 150 mL 5 mg/L RhB aqueous solution, distance 25 cm, 175 W low pressure mercury lamp	97.96% (120 min)	This work
Precipitation method combined with high-temperature calcination process	Zn(NO_3_)_2_·6H_2_O,HMT, anhydrous ethanol	0.100 g T500-ZnO nanoparticles, 150 mL 10 mg/L RhB aqueous solution, distance 25 cm, 175 W low pressure mercury lamp	92.32% (120 min)	This work
Precipitation method combined with high-temperature calcination process	Zn(NO_3_)_2_·6H_2_O,HMT, anhydrous ethanol	0.100 g T500-ZnO nanoparticles, 150 mL 15 mg/L RhB aqueous solution, distance 25 cm, 175 W low pressure mercury lamp	70.84% (120 min)	This work
Precipitation method combined with high-temperature calcination process	Zn(NO_3_)_2_·6H_2_O,HMT, anhydrous ethanol	0.100 g T500-ZnO nanoparticles, 150 mL 20 mg/L RhB aqueous solution, distance 25 cm, 175 W low pressure mercury lamp	60.19% (120 min)	This work
Precipitation method combined with high-temperature calcination process	Zn(NO_3_)_2_·6H_2_O,HMT, anhydrous ethanol	0.100 g T500-ZnO nanoparticles, 150 mL 10 mg/L RhB aqueous solution, distance 25 cm, 175 W low pressure mercury lamp	92.32% (120 min)	This work
Precipitation method combined with high-temperature calcination process	Zn(NO_3_)_2_·6H_2_O,HMT, anhydrous ethanol	0.100 g T700-ZnO nanoparticles, 150 mL 5 mg/L RhB aqueous solution, distance 25 cm, 175 W low pressure mercury lamp	99.88% (120 min)	This work
Precipitation method combined with high-temperature calcination process	Zn(NO_3_)_2_·6H_2_O,HMT, anhydrous ethanol	0.100 g T700-ZnO nanoparticles, 150 mL 10 mg/L RhB aqueous solution, distance 25 cm, 175 W low pressure mercury lamp	99.12% (120 min)	This work
Precipitation method combined with high-temperature calcination process	Zn(NO_3_)_2_·6H_2_O,HMT, anhydrous ethanol	0.100 g T700-ZnO nanoparticles, 150 mL 15 mg/L RhB aqueous solution, distance 25 cm, 175 W low pressure mercury lamp	72.08% (120 min)	This work
Precipitation method combined with high-temperature calcination process	Zn(NO_3_)_2_·6H_2_O,HMT, anhydrous ethanol	0.100 g T700-ZnO nanoparticles, 150 mL 20 mg/L RhB aqueous solution, distance 25 cm, 175 W low pressure mercury lamp	60.12% (120 min)	This work
Precipitation method combined with high-temperature calcination process	Zn(NO_3_)_2_·6H_2_O,HMT, anhydrous ethanol	0.100 g T800-ZnO nanoparticles, 150 mL 10 mg/L RhB aqueous solution, distance 25 cm, 175 W low pressure mercury lamp	44.04% (120 min)	This work
-	-	0.100 g CM-ZnO nanoparticles, 150 mL 10 mg/L RhB aqueous solution, distance 25 cm, 175 W low pressure mercury lamp	88.38% (120 min)	This work

## Data Availability

Data are contained within the article and are also available from the first corresponding author.

## Sample Availability

Samples of the compounds are available from the first corresponding author.
